# Spontaneous Coronary Artery Dissection: A Narrative Review of Epidemiology and Public Health Implications

**DOI:** 10.3390/medicina61040650

**Published:** 2025-04-01

**Authors:** Patrick Pender, Mithila Zaheen, Quan M. Dang, Viet Dang, James Xu, Matthew Hollings, Sidney Lo, Kazuaki Negishi, Sarah Zaman

**Affiliations:** 1 Westmead Applied Research Centre, Faculty of Medicine and Health, University of Sydney, Sydney, NSW 2050, Australia; 2 Department of Cardiology, Liverpool Hospital, Sydney, NSW 2170, Australia; 3 Department of Cardiology, Westmead Hospital, Sydney, NSW 2145, Australia; 4 Faculty of Medicine, University of NSW, Sydney, NSW 2033, Australia; 5 Sydney School of Health Sciences, Faculty of Medicine and Health, University of Sydney, Sydney, NSW 2050, Australia; 6 The Ingham Institute for Applied Medical Research, Sydney, NSW 2170, Australia; 7 The Victor Chang Cardiac Research Institute, Darlinghurst, NSW 2010, Australia

**Keywords:** acute coronary syndrome, spontaneous coronary artery dissection, epidemiology, public health, women, sex

## Abstract

Spontaneous coronary artery dissection (SCAD) is an uncommon but significant cause of acute coronary syndrome (ACS), predominantly affecting younger women without traditional cardiovascular risk factors. SCAD is defined as a non-atherosclerotic, non-traumatic dissection of the coronary artery, leading to the formation of an intramural haematoma or intimal tear causing obstruction to blood flow and myocardial ischaemia. Unlike traditional atherosclerotic coronary artery disease, SCAD has unique pathophysiological mechanisms. SCAD is thought to arise secondary to a bleed and/or dissection within the arterial wall, linked to hormonal influences with potential triggers of physical or emotional stress and predisposition such as an underlying connective tissue disorder. Despite being increasingly recognised, SCAD remains underdiagnosed, and knowledge regarding SCAD epidemiology is limited. In addition, the impact of SCAD extends beyond the immediate cardiac event, encompassing psychological distress, the need for rehabilitation, and long-term surveillance. This has implications not just for the patient but also their family and the healthcare system. This narrative review summarises the current knowledge of SCAD epidemiology, including the affected population, its associated risk factors, and healthcare impact. By identifying current gaps in knowledge, this review aims to encourage targeted research, public awareness, and policy initiatives to improve outcomes for individuals affected by SCAD.

## 1. Introduction

Spontaneous coronary artery dissection (SCAD) is an important but often under-recognised cause of acute coronary syndrome (ACS). It is defined as a non-atherosclerotic, non-iatrogenic, non-traumatic dissection of the coronary artery, leading to the formation of an intramural haematoma or an intimal tear causing subsequent luminal narrowing or occlusion [1,2], compromising coronary perfusion [3]. While SCAD was historically considered a rare condition, its recognition has increased substantially in recent years. This is likely due to improved imaging techniques and greater awareness among healthcare professionals [1]. Unlike atherosclerotic coronary artery disease (CAD), SCAD primarily affects younger individuals without traditional cardiovascular risk factors. SCAD further affects a predominantly female (up to 90%) population [4]. It accounts for a significant proportion of acute coronary syndromes (ACSs), particularly in young and middle-aged women, with strong associations with pregnancy, fibromuscular dysplasia (FMD), and connective tissue disorders [1,3,4,5].

There are unique challenges in the diagnosis of SCAD. This is due to the diagnosis being made on pattern recognition at the time of coronary angiography, coupled with a high clinical suspicion for SCAD. People with SCAD tend to receive similar treatment given to people with atherosclerotic ACS. However, these conventional treatments have been largely untested in people with SCAD and may have harmful effects [6]. SCAD survivors also require screening for, and ongoing surveillance of, associated vascular disorders such as fibromuscular dysplasia (FMD).

Despite increasing recognition of SCAD, the true healthcare burden of SCAD remains largely unknown. This is due to the limited global epidemiological data, particularly in low- and middle-income countries (LMICs) and the absence of dedicated global registries. This narrative review aims to explore the incidence and prevalence of SCAD and identify demographic, ethnic, and geographic variability. This was achieved through the synthesis of data from prospective and retrospective cohort studies and registries. The number of SCAD registries has expanded significantly in the past few years, with previous narrative reviews providing insights into the clinical characteristics, diagnosis, and management of SCAD [2,3]. This narrative review provides an updated and comprehensive synthesis of the epidemiological data and uniquely integrates a discussion on the healthcare costs and public health implications of SCAD areas less extensively covered in prior reviews. Healthcare challenges that arise from SCAD include difficulties in diagnosis, large knowledge gaps in management, and the lack of long-term prospective data on outcomes. By synthesising current knowledge and identifying gaps in the global reporting of SCAD, this review aims to guide future research.

## 2. Pathophysiology of SCAD

The pathophysiology of SCAD involves the formation of a false lumen within the coronary arterial wall. This is due either to an intimal tear that allows blood to enter the vessel wall [the ‘inside-out’ model] or a spontaneous rupture of the vasa vasorum leading to intramural haematoma (IMH) formation [‘outside-in’ model] [7,8,9]. The resultant intramural pressure compresses the true lumen, leading to impaired coronary perfusion and myocardial ischaemia. This process is distinct from atherosclerotic CAD, where plaque rupture or plaque erosion leads to thrombus formation within the coronary lumen [1].

The precise order of these proposed sequences of events, whether an intimal tear initiates the haematoma or whether a primary haematoma subsequently causes an intimal tear, remains an area of debate. Studies from intracoronary imaging and coronary histology endorse the ‘outside-in’ model, as up to two-thirds of SCAD cases have no identifiable communication between the arterial wall and lumen (i.e., no intimal tear). This suggests that the inciting event arose within the vessel wall [10,11]. Serial angiographic studies further support this hypothesis by demonstrating that IMH precedes intimal dissection [7]. Both mechanisms are conceivable, with the overall body of evidence indicating that either could take place, depending on the clinical context [1,12]. There is also the potential that SCAD may in fact represent two distinct, albeit closely related, entities. This uncertainty in the primary underlying mechanism adds complexity to understanding SCAD’s pathogenesis and carries implications for its diagnosis and management.

### SCAD Types

SCAD is categorised into different angiographic subtypes according to the Yip-Saw Classification (Types 1 to 4, detailed in Table 1) [13], which distinguishes them based on their angiographic characteristics and the specifics of the dissection. These classifications are important in the diagnosis of SCAD and play a role in informing treatment approaches.

## 3. Epidemiology of SCAD

### 3.1. Overall Incidence and Prevalence

Spontaneous coronary artery dissection (SCAD) is an uncommon and historically under recognised cause of acute coronary syndrome (ACS) [7]. Estimating its true global incidence remains challenging due to multiple factors including under-diagnosis, limited physician awareness, and variability in diagnostic capabilities, particularly in low- and middle-income countries [1]. Current estimates of SCAD incidence vary across different populations with significant sex-specific trends and considerable regional and ethnic variability. Overall, studies indicate that SCAD accounts for approximately 1–4% of all ACS cases [15,16,17,18,19]. Given the high burden of ACS (e.g., an estimated 805,000 ACS cases annually in the United States alone [20]), SCAD represents a significant global burden.

### 3.2. Female Sex and Young Age Predominance

While SCAD can occur in either sex and across all age groups, it most characteristically affects young and middle-aged individuals, with an average age of 51 years and an 80–90% female predominance [4]. The sex and ethnic breakdown of major SCAD studies are shown in Table 2. A female predominance of SCAD appears consistent across the world. In the large Canadian SCAD registry (*n* = 1173, prospective recruitment from 22 sites across Canada and the United States), females accounted for 89.5% of SCAD patients. In the Australian–New Zealand registries (*n* = 505, retrospective and prospective recruitment of ACS-related SCAD from 23 hospital sites), females accounted for 88.7% of the total cohort [21]. The multi-centre Mayo Clinic SCAD Registry (*n* = 1196 retrospectively recruited) demonstrated a high female predominance at 95.6% [22]. A notable exception to this female dominance was a study from the Persian Gulf area (*n* = 83, retrospective recruitment from four Gulf countries), where only 50.6% of SCAD patients were female [23]. However, this was likely due to a lack of core laboratory adjudication of SCAD in this study, whereby atherosclerotic or iatrogenic-related dissection may have been included, skewing the population to a male-predominant one.

SCAD can also occur in males, at a rate of approximately 10% of SCAD patients [6,15]. In the large Canadian SCAD registry (*n* = 1173, prospectively recruited patients, across 22 sites in North America), male prevalence was 10.5%. Males were also found to present at a younger age (mean age 49.4 ± 9.6 years vs. 52.0 ± 10.6 years in females) and were less likely to have underlying fibromuscular dysplasia (FMD) (27.8% vs. 52.7%; *p* = 0.001), depression (9.8% vs. 20.2%; *p* = 0.005), or high perceived emotional stress (3.5% vs. 11.0% *p* < 0.05) but were more likely to report isometric physical stress as a trigger for their SCAD events (25.6% vs. 7.1% *p* < 0.001) [34]. While there was no significant difference in the distribution of SCAD angiographic types between the sexes, men were more likely to have dissections involving the left circumflex artery (44.4% vs. 30.9%, *p* = 0.001) and less likely to have right coronary artery involvement (11.8% vs. 21.7%, *p* = 0.005). These findings contrast with two earlier, smaller registry studies from the USA—including a retrospective single-centre study (*n* = 87) and a retrospective analysis of a prospectively populated database (*n* = 113)—which reported an even distribution of SCAD across the coronary arteries [4,24].

Despite variable reporting of the sex differences in SCAD presentation and affected vessels, no significant sex difference has been seen in major adverse cardiovascular events (MACEs) [24].

### 3.3. SCAD in Pregnancy

SCAD is the leading cause of pregnancy-related myocardial infarction and contributes to 5–10% of maternal cardiovascular-related mortality [8,34]. In a case series of 150 patients (compiled from the published literature, conference abstracts, and institutional cases), SCAD was implicated in 43% of myocardial infarction in pregnant women. Several studies have described a heightened risk of SCAD in multiparous women (particularly more than four pregnancies) [1,35,36,37]. For example, the Mayo Clinic registry (*n* = 323 women) reported higher rates of multiparity among pregnancy-associated SCAD patients compared to both non-pregnancy SCAD cohorts and the general population (91% vs. 76% respectively, *p* = 0.018) [38]. This association was further supported by a subsequent recent meta-analysis (28 studies, *n* = 103 patients) by Apostolović et al. [36], suggesting that cumulative haemodynamic and hormonal exposures across multiple pregnancies may contribute to arterial vulnerability, although pooled statistical estimates for multiparity were not reported explicitly.

Compared to their non-pregnant counterparts, patients with pregnancy-related SCAD have been seen to have a higher rate of ST elevation MI (STEMI), left ventricular dysfunction, and cardiogenic shock [8,38]. Pregnancy-related SCAD has been associated with a more proximal coronary involvement and a more severe disease phenotype. In a single-centre study conducted by the Mayo Clinic group [38] involving 323 patients (including 54 women with pregnancy-related SCAD, retrospective and prospective recruitment), pregnancy-related SCAD was significantly more likely to involve the left main coronary artery (24% vs. 5%; *p* < 0.0001) and present as multivessel SCAD (33% vs. 14%; *p* = 0.0027) compared to non-pregnancy-related SCAD [38]. Furthermore, patients with pregnancy-related SCAD were also significantly younger (mean age 35 years vs. 47 years) and exhibited a higher incidence of ST-segment elevation myocardial infarction (STEMI) (57% vs. 36%; *p* = 0.009) and left ventricular ejection fraction ≤35% (26% vs. 10%; *p* = 0.0071). These results were similar to those of a subsequent meta-analysis (28 studies, *n* = 103 patients), which reported that patients with pregnancy-related SCAD were 30 times more likely to experience STEMI and 14 times more likely to have involvement of the left main coronary artery [36]. In addition, differences in treatment approaches have been observed, with pregnancy-related SCAD patients less likely to be managed conservatively compared to those with non-pregnancy-related SCAD, with a pooled odds ratio of 0.61 (95% CI: 0.37–0.98) [36]. This likely reflects the underlying differences in presentation of pregnancy-related versus non-pregnancy-related SCAD. Despite these differences, there was no significant difference in the SCAD recurrence rates between the two groups [36,38].

### 3.4. Regional Variation and Ethnicity

Higher incidences of SCAD have been reported in North America [15] and Europe [39], likely attributable to increased awareness and the availability of more advanced diagnostic modalities in these regions. The Canadian SCAD registry (*n* = 1173, prospective recruitment) reported that 87.3% of SCAD patients were of Caucasian descent (4.4% East Asian, 2.4% South Asian, 1.6% African Canadian, and 1.3% First Nation peoples) [7], which is disproportionate compared to their general population where approximately 70% are Caucasian [11]. SCAD has been described in Asian populations with two SCAD cohorts from China [33] and Japan [18]. The Chinese cohort (*n* = 118) was examined in a retrospective, single-centre study, whereas the Japanese cohort (*n* = 63) was part of a retrospective, multi-centre study. Both studies consisted of participants entirely from their respective national populations. In the Australian–New Zealand SCAD Registry [29] (*n* = 505 patients, prospective and retrospective recruitment), ethnicity was more rigorously collected than in past studies. The ethnic proportions in patients with SCAD more closely resembled the general population: 78.6% Caucasian, 5% East/South Asian, 1% Aboriginal or Torres Strait Islander People, 5% Māori, 1.3% Pacific Islander, and 9.6% Other [6] (see Table 2). Although there remains a paucity of data on the occurrence of SCAD among Black and Indigenous populations worldwide, it is unclear if this represents under-reporting or a true ethnic variance. Data on SCAD in populations from low- and middle-income countries (LMICs) are similarly sparse, likely related to the limited access to coronary angiography and advanced imaging techniques [40]. While true ethnic variability in SCAD may be present, the lack of reporting of ethnicity in studies and limited awareness and diagnostic capacity are critical factors contributing to the differences across ethnicities and regions.

## 4. Risk Factors and Associated Conditions with SCAD

### 4.1. Female Sex Hormones

A notable female predominance exists in SCAD, yet it is unclear whether this arises primarily from hormone-related factors or from a more complex interplay of sex- and gender-based variables—such as nonhormonal physiological differences, psychological stressors, and coexisting conditions (such as FMD). Oestrogen may influence vascular processes such as angiogenesis, vasodilation, and autonomic regulation [41,42]. Recent studies have demonstrated intrinsic sex-based differences in the arterial wall architecture between males and females and postulate that these may be contributory for SCAD [43]. Outside of pregnancy, there are reports that exogenous hormone exposure might affect this condition in particular, when used in contraception, assisted reproductive technologies, breast cancer treatments, pregnancy termination, and hormone replacement therapy during perimenopause [44].

### 4.2. Genetic Predisposition

Evidence supports a potential predisposition to SCAD within families, with a higher incidence of the condition and other associated arteriopathies among first-degree relatives of affected individuals [32,45]. Despite these reports, true hereditary clustering appears rare (approximately 1% of cases), and most evidence suggests that SCAD vulnerability is driven by multiple genetic factors rather than a single dominant mutation as no large pedigrees with multigeneration inheritance have been described [46,47,48].

Emerging data suggest that certain low-frequency allele variants may be contributory to SCAD, although not pathogenic. In one study, individuals with SCAD carried higher rates of rare fibrillar collagen gene variants, yet the clinical yield of genetic testing remains limited [1,5]. While no SCAD-specific genetic testing is currently available, SCAD has been associated with various heritable conditions, most frequently in the context of collagen type III deficiency (COL3A1). SCAD presentations have also been observed in patients with Ehlers–Danlos syndrome, Marfan syndrome (FBN1), and Loeys–Dietz syndrome (TGFBR1, TGFBR2, SMAD2, SMAD3, TGFB2, TGFB3), usually when advanced vascular abnormalities are already evident [49,50]. Furthermore, studies have linked SCAD to rare deleterious variants of thoracic-aortic aneurysm-associated genes, (e.g., LOX and FLNA), as well as in genes related to monogenic polycystic kidney disease (PKD1) [47].

In a genome-wide analysis looking at more common genetic variants, an allele in the PHACTR1 (phosphatase and actin regulator 1) common genetic locus on chromosome 6p24 (rs9349379-A) was implicated in susceptibility to SCAD [46,47,51,52]. Interestingly, this allele was also associated with FMD, which may contribute to the association between these two conditions. A recent genome-wide association meta-analysis identified 16 risk loci for SCAD, and these loci were most enriched in vascular smooth muscle cells and arterial fibroblasts [47]. Furthermore, these same loci demonstrated genome-wide negative correlation with atherosclerotic coronary artery disease. The Mayo Clinic SCAD registry discovered that approximately 45% of SCAD cases were idiopathic with no identifiable cardiocirculatory stressor, highlighting the need for more extensive genetic research to understand the heritable aspects of SCAD [53].

### 4.3. FMD and Connective Tissue Disorders

FMD has been strongly linked to SCAD [54,55]. In a single-centre, prospective study of consecutively recruited SCAD patients (*n* = 327, Vancouver Canada), complete FMD screening was performed in 80.7% of participants, revealing FMD in 62.7% of SCAD cases and was the most observed predisposing arteriopathy [3].

SCAD and FMD share a common pathophysiological mechanism primarily involving vascular smooth muscle cells and extracellular matrix [3,51,52]. These conditions share key demographic and genetic hallmarks, including onset typically in middle adulthood, a pronounced female predominance, and a heightened risk of extra-coronary vascular abnormalities, namely, aneurysms, pseudoaneurysms, dissections, and vessel tortuosity [54,55,56]. Given these associations, comprehensive vascular imaging from head to pelvis is recommended at the time of SCAD diagnosis to detect FMD and guide management. Despite these recommendations, global screening rates for FMD in SCAD survivors remain low, with many studies reporting incomplete FMD screening [15]. In the Australian–NZ Registry, only 38.6% (*n* = 182/505) of SCAD patients underwent FMD screening [6], while in the Japanese cohort, 39.6% (*n* = 25/63) had FMD screening [18]. The importance of FMD screening in SCAD survivors is clear: a high proportion will have FMD detected, and the presence of FMD has been found to predict a higher rate of adverse cardiovascular events [6].

Other vascular conditions, such as connective tissue disorders including Ehlers–Danlos syndrome type IV, Marfan syndrome, and Loeys–Dietz syndrome, have also been epidemiologically linked to SCAD [46,51], though they are less common. Real-world registry data show that these conditions occur in approximately 5% of SCAD cases [49,57,58].

### 4.4. Associated Comorbidities

Systemic inflammatory conditions such as lupus and polyarteritis nodosa have been reported in association with SCAD, suggesting that systemic inflammation may play a role in vascular vulnerability [2,59]. While SCAD tends to occur in individuals without underlying cardiac risk factors, hypertension has been observed in a high proportion of patients with SCAD. For example, in the ANZ SCAD registry (*n* = 505 patients), 30% of patients had hypertension at baseline [6]. While the specific mechanisms linking SCAD with hypertension are not fully understood, it may reflect the higher proportion of underlying vascular conditions and the association with coronary tortuosity, also observed at high rates in SCAD [32,60,61].

Studies show that a high proportion of patients with SCAD report a history of migraines [62], with rates between 32 and 46% compared to the 24% lifetime prevalence of the general female population [63]. These data suggest a 37% higher age-adjusted annual prevalence of migraines in female SCAD patients [62]. The common link between migraines and SCAD may relate to endothelial dysfunction [64,65]. This overlap suggests that similar vascular mechanisms may contribute to SCAD, underscoring the importance of further research into these shared pathological pathways. Additionally, SCAD has been correlated with other clinical conditions, such as anxiety (32%) [66,67] and depression (20%) [25,66,67,68], with the underlying pathophysiological link less known.

### 4.5. Stress and Lifestyle Risk Factors

Precipitating stressors are reported in 66% of SCAD cases [4,69]. The most common triggers for SCAD include intense emotional stress (such as bereavement or interpersonal conflict, reported in 50–55% of cases) [4,61], extreme physical exertion [3] (29% of cases), and heavy isometric activities (9% of cases) [4]. Activities that increase intrathoracic pressure, such as valsalva-like manoeuvres (e.g., coughing or vomiting), have also been reported to contribute to the onset of SCAD [1,3,7]. Elevated catecholamine levels, often linked with stress, may increase shear stress on the coronary artery wall, leading to SCAD in susceptible individuals [8].

**Figure 1:**
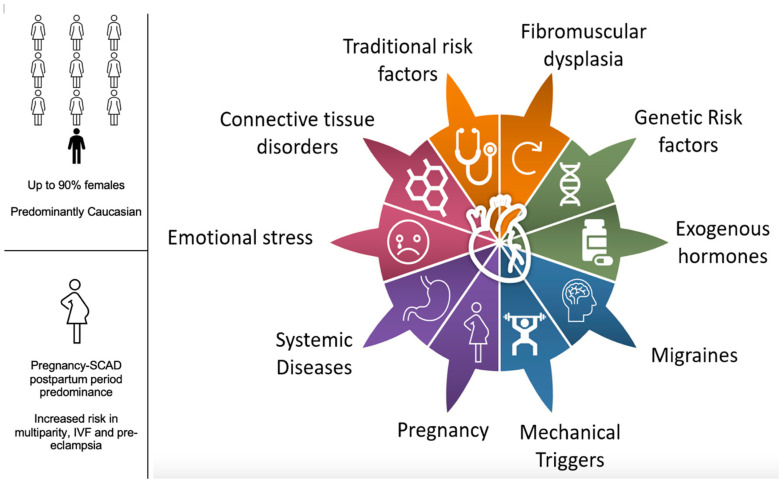
Risk factors and associated conditions with SCAD.

Interestingly, smoking poses an increased risk for SCAD, even though patients with SCAD typically present with fewer conventional risk factors compared to those with atherosclerotic coronary artery disease. Smoking is known to cause oxidative stress, which may exacerbate SCAD, but the underlying mechanism has not been elucidated. Additional lifestyle-related stressors, including substance abuse such as cocaine use [70], have been reported in patients with SCAD. Figure 1 shows an overview of risk factors and associated conditions in SCAD. 

## 5. Clinical Presentation, Diagnosis, and Management of SCAD

Substantial variations in clinical manifestations of SCAD have been seen across different demographic groups. A particularly high index of suspicion is necessary in young females presenting with ACS who lack traditional cardiovascular risk factors, particularly during pregnancy or in the postpartum period [53,71]. In the Canadian SCAD study, the majority of patients (69.9%) presented with non-ST-segment elevation myocardial infarction (NSTEMI), while 29.7% had ST-segment elevation MI (STEMI), with chest discomfort reported by 91.5% of the subjects [23]. Less common symptoms included nausea, vomiting, light headedness, and dyspnoea. A small percentage (8.1%) presented with ventricular tachycardia or fibrillation [4], and less than 1% with cardiogenic shock or sudden cardiac death [24,34]. Conversely, older patients may have coexisting atherosclerotic disease, making it more challenging to identify SCAD as the underlying cause of ACS, which can further complicate diagnosis and increase the likelihood of misdiagnosis [1,7].

### 5.1. Diagnostic Challenges and Differentiation from Other ACS Causes

The accurate diagnosis of SCAD requires a combination of clinical suspicion and advanced imaging, as differentiation from atherosclerotic ACS is crucial due to substantial differences in patient management [1]. SCAD is often underdiagnosed, partly because it occurs in individuals without typical cardiovascular risk factors and clinicians may fail to consider ACS in a young female. Diagnostic tools including invasive coronary angiography (ICA), intravascular ultrasound (IVUS), and optical coherence tomography (OCT) can be essential in differentiating SCAD from other causes of ACS. This includes differential diagnoses of atherosclerotic-related ACS, coronary vasospasm, takotsubo cardiomyopathy, myocardial bridging, and embolisation [14]. However, these imaging modalities are not universally available, particularly in resource-limited settings.

ICA remains the gold standard for SCAD diagnosis. ICA can further facilitate recognition of high-risk anatomical features such as left main, proximal vessel involvement, and/or multivessel SCAD. Angiographic features specific to SCAD include long diffuse narrowing (ranging from 45 to 48 mm—longer than those typically observed in atherosclerotic conditions [14]), a “double lumen” appearance, and/or increased vessel tortuosity defined as three or more consecutive vessel curvatures of 90–180° (see Table 1, Types of SCAD). Tortuosity is most commonly seen in the left circumflex artery and often affecting non-culprit vessels [61,71]. ICA alone, however, can sometimes be ambiguous [14,72], leading to the potential misclassification of SCAD as atherosclerotic disease [1,11].

In situations of diagnostic ambiguity, the use of advanced intravascular imaging techniques such as IVUS and OCT [10,14,73] can be invaluable in determining the presence of intimal dissection and identification of intramural haematoma, false lumen, and fenestrations. IVUS offers insights into the vessel wall’s integrity and can identify intramural haematomas, whereas OCT provides superior resolution in the imaging of the arterial layers, crucial for assessing dissection planes and intramural pathology [1,74]. The use of contrast during OCT has the potential to exacerbate dissections, leading to further vessel injury. A study of 65 patients with SCAD who underwent OCT found that 7.9% experienced an angiographic complication [10]. However, in a smaller study of SCAD patients undergoing OCT (*n* = 11), no patients experienced OCT complications [74]. Despite their utility in the diagnosis of SCAD, intravascular imaging techniques such as IVUS and OCT remain underutilised due to concerns regarding safety and cost. The limited availability of OCT and IVUS in resource-constrained settings also prevents their use in SCAD. Both modalities require specialised equipment and expertise, which may restrict their use to tertiary care centres. Consequently, the adoption of OCT and IVUS into the clinical diagnosis of SCAD remains limited [8,75], and this may contribute to the under-diagnosis of SCAD. Furthermore, current guidelines do not outline a clear diagnostic algorithm for the use of these imaging modalities [1,5]. While they suggest considering IVUS or OCT in cases of diagnostic uncertainty, they lack detailed guidance on patient selection criteria and procedural precautions to minimise the risks. This absence of specific recommendations may also contribute to their low uptake in clinical practice [1].

### 5.2. Non-Invasive Adjunctive Imaging in SCAD

Cardiac MRI and coronary CT angiography (CCTA) may play an emerging role in the evaluation of SCAD, particularly in cases where coronary angiography is inconclusive or when further assessment of extra-coronary vascular abnormalities is needed. These imaging modalities are especially helpful in identifying associated conditions such as FMD or other vascular abnormalities [76,77,78] (see Section 4.3 FMD and Other Connective Tissue Disorders).

CCTA provides a non-invasive method for coronary imaging that minimises the risk of iatrogenic dissection compared to invasive procedures. The limitations of CCTA include the potential for motion artifacts and a lower spatial resolution (approximately 0.5 mm) compared to invasive coronary angiography (0.1 to 0.2 mm). CCTA findings that have been described in SCAD include abrupt stenosis, intramural haematoma, tapered stenosis over 50%, and dissection flaps. However, specific CT diagnostic criteria in SCAD are not clearly defined [73,78], and CCTA cannot conclusively differentiate between atherosclerotic disease and intramural haematoma or exclude SCAD in more distal vessels [79]. Furthermore, in a small study (*n* = 11 patients, 18 lesions) that compared invasive angiography with CCTA in patients with confirmed SCAD, CCTA missed 20% of the SCAD cases, mostly due to the lack of detection of SCAD in distal segments [80]. CCTA may be more useful as an adjunct to invasive coronary angiography. In this instance, CCTA can be used to exclude extensive atherosclerosis or to demonstrate healing in larger or more proximal vessel SCAD.

Cardiac magnetic resonance (CMR) has a limited but emerging role in the diagnosis of SCAD. Currently, its main role is in the confirmation of myocardial infarction through late gadolinium enhancement to show the territory of infarction. This in turn may lead the clinician to identify more subtle SCAD in vessels supplying that territory. CMR may also aid in the assessment of left ventricular ejection fraction and therefore help to predict outcomes [81]. Magnetic resonance angiography can be useful in assessing FMD, particularly when further CT scans may be contraindicated.

Multi-modality imaging also plays a role in the follow-up of SCAD survivors. Around one-third of patients with SCAD report recurrent chest pain, which may be due to a variety of causes including further SCAD episodes, coronary microvascular and endothelial dysfunction, or non-ischaemic factors [8]. When chest pain continues in the absence of recurrent ACS, non-invasive diagnostic methods such as quantitative perfusion cardiac magnetic resonance imaging and positron emission tomography may be useful for detecting coronary microvascular disease. CCTA can help to demonstrate healing and exclude a more proximal, non-healed SCAD-related stenosis that could be contributing to pain. Continued research is essential to fully understand the incidence and nature of coronary microvascular disease following SCAD and to investigate the non-ischaemic factors that contribute to persistent chest pain in these patients.

### 5.3. Impact of Accurate Diagnosis on Management in SCAD

The importance of distinguishing SCAD from other forms of ACS cannot be overstated, as it impacts both treatment and outcomes. The therapeutic approach for SCAD differs significantly from that of atherosclerotic ACS. Conservative management is preferred, especially in stable patients, due to the risk of procedural complications during percutaneous coronary intervention (PCI). Furthermore, the use of antiplatelet therapy in SCAD remains under debate. Dual antiplatelet therapy (DAPT), currently the standard of care for atherosclerotic ACS, may not be beneficial in SCAD, with two recent studies demonstrating the potential for harm, particularly with more potent P2Y12 inhibitors [30,82,83] [Table 3]. The use of statins is also not routinely indicated unless there are concomitant atherosclerotic risk factors. Beta-blockers have been associated with lower rates of SCAD recurrence and are recommended for all patients with SCAD (Table 3) [3,83,84].

Proper diagnosis allows for tailored counselling regarding the aetiology, prognosis, and risk of recurrence in SCAD. While some SCAD cases appear linked to genetic factors—particularly connective tissue disorders such as Ehlers–Danlos and Marfan syndromes—comprehensive genetic testing for all patients has been found to yield relatively low returns overall and is therefore not routinely recommended [85,86]. Nonetheless, targeted genetic evaluation may be considered in specific circumstances, such as recurrent SCAD, where extra-coronary vascular anomalies are present, in family clusters of SCAD, or where there is a known hereditary connective tissue condition in a first-degree relative [86]. During clinical assessment, signs of connective tissue disease should be screened and assessed for, e.g., joint hypermobility, skin hyperextensibility, arachnodactyly, or lens subluxation (ectopia lentis). Echocardiography may also help detect valvular abnormalities associated with connective tissue disorders. In patients where genetic testing is deemed appropriate, gene panels focusing on aortopathies and connective tissue syndromes are often employed. In these instances, structured genetic counselling for both the patient and family members is an essential component of care.

**Table 3:** Comparison of the management of acute coronary syndrome caused by spontaneous coronary artery dissection vs. atherosclerosis.

Management Strategy	Atherosclerotic ACS [87,88]	SCAD ACS [1,5]
Antiplatelets	Dual antiplatelet therapy (DAPT) for up to 12 months [In non-high-bleeding-risk patients]. Continue aspirin long term.	DAPT if stenting was performed. In conservatively managed patients, at least one antiplatelet (preferably aspirin) with duration of therapy not established.
Anticoagulation	Parenteral anticoagulation is recommended for all patients at the time of diagnosis.	Not recommended unless there are other indications.
RAAS system inhibitors	Recommended in patients with heart failure symptoms, LVEF ≤ 40%, diabetes, hypertension, or chronic kidney disease.	Recommended in patients with heart failure symptoms, LVEF ≤ 40%, or hypertension.
Beta-blockers	Recommended in patients with LVEF ≤ 40%.	Recommended in all patients to reduce the risk of SCAD recurrence
Statins	High-dose statin therapy recommended for all patients.	Not recommended unless there are other indications.
Cardiac rehabilitation	Recommended for all patients.	Recommended for all patients.
Fibromuscular dysplasia screening	Not recommended.	Recommended for all patients.

### 5.4. SCAD Management

Two consensus statements published in 2018 from the American College of Cardiology (ACC)/American Heart Association (AHA) and the European Society of Cardiology (ESC) supported a conservative approach (i.e., no revascularisation) for stable patients with SCAD [1,5]. The rationale for a conservative approach was due to there being a high likelihood of SCAD healing over time, with studies showing complete resolution in more than 90% of cases by 30 days [72,89]. In addition, PCI was associated with high rates of complications, including slow/no flow, iatrogenic dissection, and long-term stent malposition. In a proportion of patients with SCAD, however, urgent revascularisation with PCI or coronary artery bypass grafting is required. This may include the presence of high risk anatomy (left main, double proximal vessel disease) and/or haemodynamic instability with cardiogenic shock and/or ongoing ischaemia [1,5]. There remains an urgent need for further data to inform management strategies for patients with SCAD.

In large cohort studies, the rate of MACEs on long-term follow-up was between 11.3% and 14%, with mortality from 0.7% to 1.6% [6,15]. The mortality rate of ACS caused by SCAD was generally lower than atherosclerotic ACS, where an approximate 3% mortality over three years has been described [90,91,92]. The three-year rate of SCAD recurrence has further been described as occurring in 2.5% to 5.7% of patients [6,15]. The recurrence rate by different populations has not been reported.

The long-term management of SCAD emphasises cardiac rehabilitation and psychosocial support to improve patient outcomes and quality of life. Cardiac rehabilitation is recommended for all SCAD patients, with existing data suggesting that tailored, low- to moderate-intensity aerobic exercise is safe and beneficial [93]. Activities such as walking, cycling, and swimming are often encouraged, while high-intensity or isometric resistance exercises are generally avoided due to hypothetical concerns about arterial stress and recurrent SCAD, although evidence for such strategies is lacking [1,94,95,96,97]. Further studies are needed to provide clearer guidelines on exercise for SCAD survivors. It is likely that the physiological and psychosocial benefits of regular, moderate-intensity activity outweigh the theoretical risks [8,93]. However, the uncertainty surrounding appropriate exercise regimens can contribute to anxiety and reluctance to engage in physical activity in SCAD survivors, underscoring the need for more clear guidance [93,98].

Psychosocial support is equally critical in the long-term care of SCAD patients, with more than one in four experiencing anxiety or depression, primarily driven by a fear of recurrence or exercise resumption [99,100]. Psychological interventions, including counselling, cognitive-behavioural therapy, and stress management techniques, have been used to help patients cope with the emotional impact of their diagnosis [1,53,101]. Integrating psychosocial care into cardiac rehabilitation programmes may enhance mental well-being and promote adherence to rehabilitation.

The long-term management of SCAD involves screening for the related condition FMD [Table 3] [1,5,8] (see Section 4.3 and Section 5.2 for details).

## 6. Public Health Implications and Future Directions

### 6.1. Healthcare Costs

The economic impact of SCAD represents a significant, yet often underexplored, facet of its overall healthcare burden. In one study in the United States on 31,105 patients with SCAD between 2016 and 2020, the mean cost of hospitalisation was USD 22,470 [102]. In comparison, the average cost of admission for acute coronary syndrome in 2009 in the USA was USD 63,578 [103]. The healthcare cost of SCAD includes not only the direct costs associated with hospitalisation, advanced imaging, interventions, medications, and specialist consultations but also considerable indirect costs. These indirect costs arise from the diminished quality of life and mental health challenges that can affect a survivor’s ability to resume normal activities or maintain employment. Additionally, the psychological effects of SCAD, such as persistent anxiety and fear of recurrence, often extend recovery periods and reduce productivity, thereby amplifying the economic burden on both patients and the healthcare system [7].

Hospitalisations for SCAD typically involve extended stays in coronary or intensive care units, with reported durations ranging from 5 to 7 days, which is notably longer than the stays for atherosclerotic ACS [7,8]. Unlike atherosclerotic ACS patients who often undergo rapid revascularisation and discharge, SCAD patients are primarily managed medically, potentially necessitating repeat invasive angiography to monitor healing [8]. This care approach, coupled with the absence of standardised treatment pathways distinct from those for atherosclerotic ACS, can lead to protracted hospital stays and increased healthcare expenditures. Moreover, the likelihood of misdiagnosis is higher in SCAD due to the typical patient profile being younger and female, leading to additional diagnostic procedures and longer hospital stays [1].

Complications such as cardiac arrest and heart failure have been described to occur in 1.2% and 28% of patients with SCAD, respectively [6]. These contribute to healthcare costs, related to medical therapy, specialist follow-up, and potential device implantation. Over 50% of SCAD survivors experience recurrent chest pain syndromes, leading to frequent emergency department visits, hospital admissions, repeated imaging, and cardiology consultations [1]. The emotional and psychological impact of SCAD may be considerable, with 20–30% of SCAD patients reporting preexisting mental health conditions [22] and SCAD survivors also reporting high proportions of anxiety, depression, and post-traumatic stress disorder (PTSD) [104]. These challenges can affect quality of life and lead to increased healthcare utilisation. As patients with SCAD are frequently young (average age of 51 years), the loss of quality-adjusted life years (QALY) is particularly significant, emphasising the need for comprehensive approaches that include both medical and psychological support to manage and mitigate the extensive healthcare costs associated with SCAD.

### 6.2. Considerations for Healthcare Policy

The public health implications for SCAD should focus on raising awareness and enhancing education around this condition. For healthcare professionals, enriched training on recognising and managing SCAD is crucial to decrease misdiagnosis rates and ensure that patients receive appropriate and prompt care. Furthermore, from an equity standpoint, it is crucial to acknowledge that SCAD is likely underdiagnosed in low-resource settings. Embedding SCAD into regular cardiovascular education through targeted programmes and workshops can help close existing knowledge gaps and equip professionals to better handle these cases.

Raising awareness among the general public, especially within high-risk groups, is critical for early detection and timely medical intervention, potentially leading to improved patient outcomes [8,105]. Moreover, incorporating mental health services into SCAD patient care is essential to address the significant psychological impact associated with this condition, which could lead to better overall health outcomes and lower long-term healthcare usage [3,7]. Policy initiatives that support multidisciplinary teams, including cardiologists, mental health specialists, and rehabilitation experts, are vital for delivering comprehensive care [1].

Supporting the families of SCAD patients is also crucial, as the effects of the condition often extend beyond the patients to their caregivers, who may experience considerable emotional and financial strain [8]. Providing access to educational resources, psychological support, and social services may help ease this burden [3,69]. Additionally, workplace policies that support extended leave and flexible work arrangements for SCAD patients may reduce the economic impact on families and the broader workforce [1].

Ultimately, healthcare policies could play a central role in enhancing the quality of life for individuals affected by SCAD and in reducing the broader economic burden on healthcare systems. Policymakers and healthcare providers could prioritise the availability of resources used in the diagnosis of SCAD (such as invasive angiography and adjunctive imaging) and fund rehabilitation programmes and mental health services [1].

### 6.3. Future Directions in SCAD Research

One of the current gaps in SCAD research is the lack of robust epidemiological data, particularly among individuals from diverse ethnic backgrounds. The majority of existing data are derived from studies that may not reflect the true diversity of SCAD worldwide. The current World Health Organization (WHO)’s International Classification of Disease (ICD)-10 codes do not capture SCAD as a distinct diagnosis. Global adoption of the WHO’s proposed ICD-11 codes, which include a code for coronary artery dissection (BA82), could allow a more accurate understanding of SCAD prevalence. In particular, widespread adoption of ICD-11 codes could provide a means of reporting SCAD in under-resourced settings, such as in low- and middle-income countries.

In addition to improving knowledge on the true epidemiology of SCAD, further studies are needed to guide diagnosis and management and determine outcomes. Multicentre registries with prospective recruitment of patients would aid in the collection of standardised, high-quality data from a diverse set of populations. Such registries do exist, such as the Canadian SCAD, European SCAD, and Australian–New Zealand SCAD registries [6,15,29], but more registries in more diverse settings are needed. Data pooling across these cohorts would overcome the limitations of smaller studies and potentially address the current gaps in SCAD knowledge. Additionally, more research is needed to understand the role of environmental triggers, including hormonal influences, psychological stress, and physical exertion, in precipitating SCAD. Due to improved awareness and diagnosis of SCAD, there is a growing global cohort of SCAD survivors but a lack of evidence to guide optimal long-term management. Future research should aim to develop large-scale randomised controlled trials to determine the ideal management strategies in SCAD. Further, more research is needed to guide exercise/physical activity re-engagement and mental health management, which have the potential to impact long-term outcomes for these patients.

## 7. Conclusions

SCAD is an uncommon cause of ACS that affects a younger, predominantly female, Caucasian population. The true prevalence of SCAD is likely to be an underestimation globally, due to the underutilisation of coronary angiography and adjunctive imaging modalities that can be crucial in distinguishing SCAD from atherosclerotic ACS. SCAD not only imposes an immediate healthcare burden but also profoundly affects long-term outcomes, with significant rates of major adverse events as well as an impact on quality of life and mental health conditions. There is an urgent need for more comprehensive epidemiological data in people with SCAD. This would enhance understanding, and, in turn, help optimise healthcare resource utilisation.

## Figures and Tables

**Table 1:** The Yip-Saw Angiographic Classification: phenotypic recognition of SCAD [13,14].

Type of SCAD	Description	Angiographic and Intravascular Imaging Appearance
Type 1	Classic double lumen appearance with an intimal flap, easily identifiable on imaging.	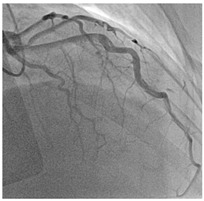 Type 1 SCAD of LAD mid-distal vessel.
Type 2a	Long, diffuse narrowing with a segment of the artery returning to normal calibre distally with no improvement following IC GTN	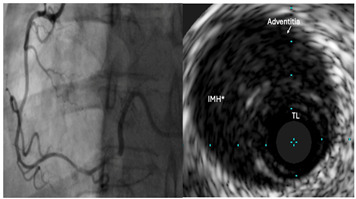 Type 2a SCAD of RCA with extension into the RPL. IVUS confirms large intramural haematoma (IMH*) with true lumen [TL] compression.
Type 2b	Long, diffuse narrowing extending to the distal end without returning to normal calibre with no improvement following IC GTN	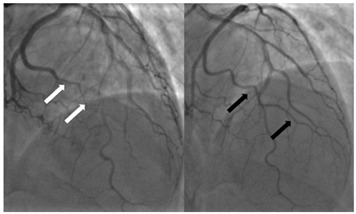 Type 2b SCAD of OM1 [initial angiogram white arrows]; repeat angiogram at 3 months with vessel healing [black arrows].
Type 3	Mimics focal atherosclerotic disease, presenting as a short, focal stenosis. Requires high index of suspicion and IVUS/OCT for diagnosis.	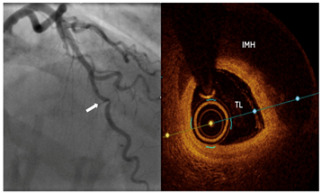 Type 3 SCAD in mid LAD (white arrow). Diagnosis confirmed on OCT imaging showing IMH causing true lumen [TL] stenosis.
Type 4	Total occlusion of the vessel often misinterpreted as thrombotic occlusion.	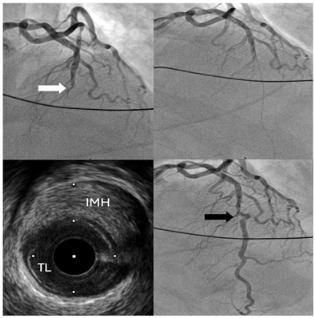 Type 4 SCAD of LAD: Coronary angiogram of mid LAD [white arrow]. IVUS performed that confirmed SCAD diagnosis with IMH. Flow restoration following successful balloon angioplasty [black arrow].

**Table 2:** Sex, ethnicity, and regional variability in SCAD.

Registry Name	Country	Cohort Size	Female (%)	Male (%)	Caucasian (%)	All Other Ethnic Groups
Canadian SCAD Study [24]	Canada	1173	89.5%	10.5%	Not reported	Not reported
Massachusetts General Hospital SCAD Registry [25]	USA	113	87%	13%	74%	4% Black 3% Asian 20% Unknown
Kaiser Permanente SCAD Study [26]	USA	111	92.8%	7.2%	50.5%	13.5%, Black 16.2% Hispanic 18% Asian 1.8% Other
Multi-centre SCAD Registry from KPSC System [21]	USA	208	88.9%	11.1	33.7%	15.9% Black 41.4% Hispanic 6.3% Asian 2.9% Other
Mayo Clinic SCAD Registry [27]	USA	1196	95.6%	3.9%	92.3%	2.3% Black 2.2% Hispanic 1.3% Asian 0.2%Asian Indian 0.4% Native American 0.1% Polynesian 0.2% Unknown
Spanish SR-SCAD Registry [28]	Spain	318	88%	12%	91%	Not reported
ANZ SCAD Registry [29]	Australia and New Zealand	505	88.7%	11.3%	78.6%	1% Aboriginal and Torres Strait Islander 4.4% Māori 1.3% Pacific islander 3.3% East Asian 1.7% South Asian 9.6% Other/Unknown (including Middle Eastern, African, North African)
DISCO IT/SPA Registry [30]	Italy and Spain	332	88.9%	11.1%	Not reported	Not reported
National French SCAD Registry DISCO [31]	France	373	90.6%	9.4%	Not reported	Not reported
UK SCAD Registry [32]	United Kingdom	170	94.2%	5.8%	94.2%	1.2% Black 2.9% Indian 1.2% Other 1% Not reported
iSCAD Registry [22]	Australia, USA	859	93.9%	6.1%	89%	7.3% Black 1.8% Asian 0.4% Native Alaskan/Native American 1% Multiracial 0.5% Other 0.1% Pacific Islander/Native Hawaiian
Japan SCAD registry [18]	Japan	63	94%	6%	0%	100% Japanese
Chinese SCAD Study [33]	China	118	14%	86%	0%	100% Chinese
Gulf SCAD Registry G-SCAD [23]	Four Gulf countries	83	50.6%	49.4%	Not reported	84% Arabic Remainder not reported

## Abbreviations

The following abbreviations are used in this manuscript:

SCAD	Spontaneous coronary artery dissection
AMI	Acute myocardial infarction
ACS	Acute coronary syndrome
ICA	Invasive coronary angiography
IMH	Intramural haematoma
FMD	Fibromuscular dysplasia
MACE	Major adverse cardiovascular event
IVUS	Intravascular ultrasound
OCT	Optical coherence tomography
CCTA	Coronary CT angiography
CMR	Cardiac magnetic resonance
